# Advanced nursing practice and interprofessional dementia care (InDePendent): study protocol for a multi-center, cluster-randomized, controlled, interventional trial

**DOI:** 10.1186/s13063-022-06249-1

**Published:** 2022-04-11

**Authors:** Fabian Kleinke, Bernhard Michalowsky, Anika Rädke, Moritz Platen, Franka Mühlichen, Annelie Scharf, Wiebke Mohr, Peter Penndorf, Thomas Bahls, Neeltje van den Berg, Wolfgang Hoffmann

**Affiliations:** 1 Institute for Community Medicine, Section Epidemiology of Health Care and Community Health, University Medicine Greifswald, Greifswald, Germany; 2 German Center for Neurodegenerative Diseases (DZNE), Site Rostock/Greifswald, Greifswald, Germany

**Keywords:** Advanced nursing practice, Advanced nursing roles, Collaborative care, Tasks, Delegation, Substitution, Alzheimer’s disease, Dementia, General practitioner, Nursing

## Abstract

**Background:**

A redistribution of tasks between specialized nurses and primary care physicians, i.e., models of advanced nursing practice, has the potential to improve the treatment and care of the growing number of people with dementia (PwD). Especially in rural areas with limited access to primary care physicians and specialists, these models might improve PwD’s quality of life and well-being. However, such care models are not available in Germany in regular healthcare. This study examines the acceptance, safety, efficacy, and health economic efficiency of an advanced nursing practice model for PwD in the primary care setting in Germany.

**Methods:**

InDePendent is a two-arm, multi-center, cluster-randomized controlled intervention study. Inclusion criteria are age ≥70 years, cognitively impaired (DemTect ≤8) or formally diagnosed with dementia, and living in the own home. Patients will be recruited by general practitioners or specialists. Randomization is carried out at the physicians’ level in a ratio of 1:2 (intervention vs. waiting-control group). After study inclusion, all participants will receive a baseline assessment and a follow-up assessment after 6 months. Patients of the intervention group will receive advanced dementia care management for 6 months, carried out by specialized nurses, who will conduct certain tasks, usually carried out by primary care physicians. This includes a standardized assessment of the patients’ unmet needs, the generation and implementation of an individualized care plan to address the patients’ needs in close coordination with the GP. PwD in the waiting-control group will receive routine care for 6 months and subsequently become part of the intervention group. The primary outcome is the number of unmet needs after 6 months measured by the Camberwell Assessment of Need for the Elderly (CANE). The primary analysis after 6 months is carried out using multilevel models and will be based on the intention-to-treat principle. Secondary outcomes are quality of life, caregiver burden, acceptance, and cost-effectiveness. In total, *n*=465 participants are needed to assess significant differences in the number of unmet needs between the intervention and control groups.

**Discussion:**

The study will provide evidence about the acceptance, efficacy, and cost-effectiveness of an innovative interprofessional concept based on advanced nursing care. Results will contribute to the implementation of such models in the German healthcare system. The goal is to improve the current treatment and care situation for PwD and their caregivers and to expand nursing roles.

**Trial registration:**

ClinicalTrials.gov NCT04741932. Registered on 2 February 2021.

## Administrative information

Note: the numbers in curly brackets in this protocol refer to SPIRIT checklist item numbers. The order of the items has been modified to group similar items (see http://www.equator-network.org/reporting-guidelines/spirit-2013-statement-defining-standard-protocol-items-for-clinical-trials/).

**Table:** 

Title {1}	Advanced nursing practice and interprofessional dementia care (InDePendent): Study protocol for a multi-center, cluster-randomized, controlled, interventional trial
Trial registration {2a and 2b}.	ClinicalTrials.gov, NCT04741932
Protocol version {3}	10.08.2021, BB144/20a
Funding {4}	The study is funded by the Innovation Fund of the Federal Joint Committee (G-BA) in the funding program “New forms of care”. Funding ID: 01NVF18034. The funding period is set between April 1, 2020 and September 30, 2023.
Author details {5a}	^1^ Institute for Community Medicine, Section Epidemiology of Health Care and Community Health, University Medicine Greifswald, Greifswald, Germany ^2^ German Center for Neurodegenerative Diseases (DZNE) e.V., site Rostock/ Greifswald, Greifswald, Germany
Name and contact information for the trial sponsor {5b}	ClinicalTrials.gov, NCT04741932
Role of sponsor {5c}	The funding body was not actively involved in the study design or will be in the data collection or analysis or interpretation or publication of the results.

## Introduction

### Background and rationale {6a}

With an increase in life expectancy and the associated increase of the number of elderly people, a rise in age-associated illnesses, such as dementia, represents a challenge for health care systems worldwide, where around 50 million people have dementia [1]. This number is expected to double every 20 years to 130 million people living with dementia (PwD) in 2050 worldwide. The increase of PwD will cause an increasing need for care, associated with a substantial increase in cost. Thus, the growing number of PwD is expected to represent a substantial social and economic burden worldwide [1–5]. To decelerate the progression of dementia diseases, PwDs depend on a timely diagnosis and evidence-based post-diagnostic care and support in line with national guidelines [6]. Despite existing evidence for treatments that can improve symptoms and delay the progression of dementia, adherence to national dementia guidelines is currently lacking. As a consequence, the majority of PwD (99%) have at least one, and most have several unmet needs of care [7–9].

Several countries have implemented collaborative care models to overcome the existing challenges of inadequate post-diagnostic support [4, 10–13]. Nurses who work in close cooperation with GPs form the central component of such models. Nurses often have a closer relationship with the patients than GPs, as they usually meet the patients more frequently and are aware about the personal living conditions. Additionally, these nurses have a clearer understanding of person-centered care [14]. In some cases, nurses take on expanded roles including tasks usually performed by GPs. Such concepts of treatment and care are generally summarized as advanced nursing practice (ANP).

Laurent et al. found that delivery of primary healthcare services by nurses instead of physicians, i.e., ANP, results in an equal or possibly better quality of care, including similar or better health outcomes for patients and higher levels of patient satisfaction [15]. Other studies confirmed that ANP- approaches are safe and can optimize treatment and care and patients’ outcomes [13, 16–20]. As a consequence, ANP was implemented years ago in several countries, like Canada, the USA, and most European countries [21]. In most countries, ANP nurses are allowed to order diagnostic tests, to diagnose independently, and to prescribe medications [22–24].

Even though ANP with various degrees of nurse autonomy is common practice [18, 19, 21, 23, 25–32], ANP with independence taking over tasks of GPs does not exist in Germany to date [22]. Within the InDePendent project, dementia care managers (DCM) will take over tasks in cooperation [33]. However, the demand for primary care treatments is expected to rise with the proceeding demographic change. There are already indications of shortages of personal resources in the health care sector. Despite a rising number of GPs over the past decade, the total number of GP hours has decreased and shortages of GPs are imminent, particularly in rural and remote areas. A redistribution of tasks between GPs and nurses provides an option to compensate for this gap [34].

Several initiatives have tried to promote structures for the implementation of ANP models [35–38] in Germany. However, due to the lack of a respective legal framework in German social law, the common practice of ANP in Germany remains theoretical. The only legally possible exception is to implement ANP as part of interventional research to evaluate its safety, effectiveness, and efficiency.

### Objectives {7}

The overall goal of the intervention is (1) to assess and address patients’ and caregivers’ unmet healthcare needs to (2) improve the health and living situation of the PwD and caregivers by providing individualized, person-centered and advanced dementia care management (DCM). In addition, the InDePendent study will examine the acceptance, safety, efficacy, and efficiency of an ANP model in primary dementia care in Germany. The aim of this study is to evaluate, whether dementia-specific qualified nurses and a redistribution of tasks between nurses and primary care physicians could significantly reduce the number of unmet needs of PwD compared to routine care after 6 months. Additionally, the study aims to improve PwDs’ quality of life and relieve the informal caregiver burden. Finally, the aim is to evaluate the acceptance and cost-effectiveness of the intervention.

### Trial design {8}

**Fig. 1:**
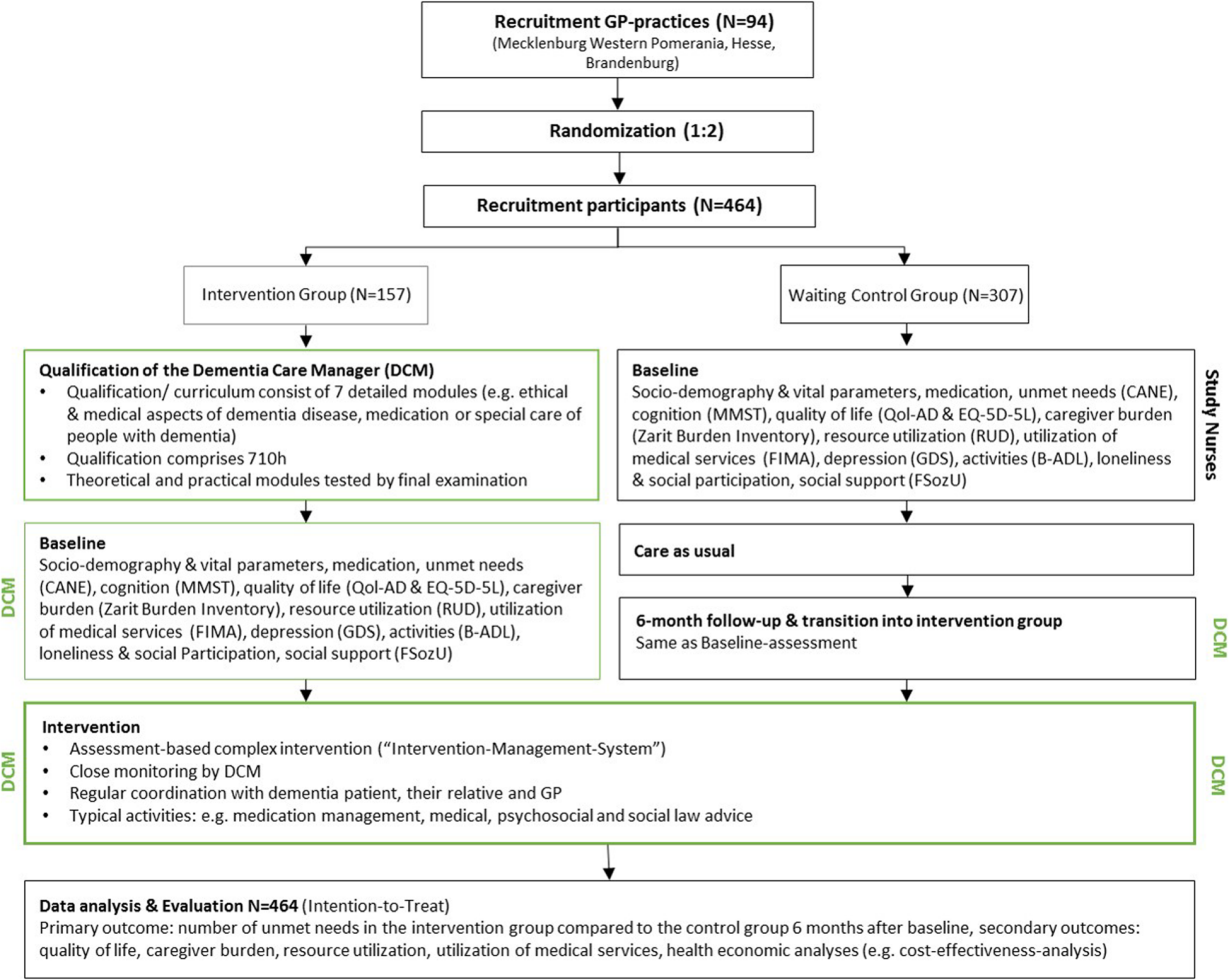
Flowchart of the InDePendent study

The InDePendent study is a multi-center, cluster-randomized, controlled intervention study with two arms: (1) patients in the intervention group will receive a model of advanced dementia care management for 6 months, and (2) a waiting-control group, which will start with the usual care for 6 months and subsequently receive the advanced dementia care management (1:2). The flow chart of the study is illustrated in Fig. 1.

## Methods: participants, interventions, and outcomes

### Study setting {9}

Study participants are recruited within primary care by GPs and specialists, e.g., neurologists and psychiatrists, who are members of five physician networks in three federal states of Germany (Mecklenburg-Western Pomerania, Brandenburg, and Hesse).

### Eligibility criteria {10}

The study population consists of people who meet the following inclusion criteria: (1) age ≥70 years, (2) DemTect Score ≤ 8 or a formal diagnosis of any with dementia by the treating GP or neurologists/ psychiatrists, (3) living in the community (i.e., at home and not institutionalized), and (4) providing written informed consent to participate in the study.

### Who will take informed consent? {26a}

PwDs and their informal caregiver will be asked for their informed consent (IC) to participate in the study. If patients are unable to provide written IC, their legal representative will be asked to provide written IC on their behalf. The GPs will be responsible to verify the patients’ ability to provide a valid IC.

### Additional consent provisions for collection and use of participant data and biological specimens {26b}

Additionally, patients of the two statutory health insurances which participate in the InDePendent study will be asked for their consent to retrieve their health insurance data.

## Interventions

### Explanation for the choice of comparators {6b}

Participants in the waiting-control group will receive care as usual in a primary care setting for 6 months. This group will serve as the comparator for the intervention. After 6 months, the participants in the waiting-control group will change to the intervention group.

### Intervention description {11a}

The DCM concept is based on a collaborative model of care that was previously implemented and evaluated within the DelPHi-MV trial [39]. The InDePendent intervention is extended by advanced nursing roles. Therefore, the intervention will be provided by dementia-specifically-qualified nurses, who will take on particular tasks, that are usually performed by primary care physicians. The DCM qualification was based on curricular guidelines of the Federal Joint Committee on the definition of medical tasks that can be transferred to nurses (§63 Para. 3c of the Social Law Book XI in Germany). The developed curricula were evaluated and approved by the Ministry of Health and the Ministry of Families and were used for the first time to enhance nurses’ competencies to overtake tasks of GP in dementia care. A close cooperation between the GP and the DCM is an important part of the intervention. The intervention is multidimensional and will be individually tailored to the patients’ and caregivers’ specific healthcare needs and their social contexts and resources.

In detail, the intervention consists of four main parts: (1) assessment of patients and caregivers’ unmet medical, pharmaceutical, psychosocial, social, and nursing care needs; (2) development of a plan for needs-based activities; (3) implementation of the intervention tasks needed to address unmet needs; and (4) monitoring of the intervention. The intervention is based on the three principles: (i) management of treatment and care, (ii) medication management, and (iii) caregiver support.

The intervention will be facilitated by a developed computerized intervention management system (IMS) to support the nurses in identifying patients’ and caregivers’ unmet needs and in the development and monitoring of the intervention care plan. The rule-based expert decision support system uses predefined algorithms based on special items within questionnaires and automatically identifies unmet needs and subsequently suggests the corresponding specific treatment and care options to address the identified unmet needs. Therefore, individual patient characteristics were matched to a computerized knowledge base. The IMS system was previously used within the dementia care management of the DelpHi-MV trial, providing evidence that IMS improves the systematic identification of unmet needs and the subsequent recommendation of interventions to address these needs [40].

Therefore, the collaborative model of care (multi-component intervention) will be delivered according to a detailed protocol. The specialized nurse will meet the PwD and their caregiver at their homes for the comprehensive assessment. During the first month of the 6 months intervention, the digitally developed detailed and individual intervention plan will be discussed between the specialized nurse and the GP or specialists, to determine which tasks will be carried out by the specialized nurse and by the physician, respectively.

There are three types of tasks to be carried out by the specialized nurse: (a) interventions after consultation with the GP or specialist (delegation), (b) interventions without prior GP-consultation provided autonomously by the specialized nurse (substitution), and (c) recommendations by the specialized nurse that have to be carried out by the GP or specialist (cooperation).

In addition to the mandatory contact between the nurses and GPs in the first month of intervention, additional contacts after three months are optional, depending on the specialized nurses’ or practitioners’ individual needs and preferences. At the end of the intervention after 6 months, the practitioners receive a comprehensive GP letter with information on the health situation of the patient and recommendations for further management.

### Criteria for discontinuing or modifying allocated interventions {11b}

The intervention will be implemented individually according to patients’ and caregivers’ unmet needs. However, the intervention will be discontinued or modified if:

- The patient moves to a nursing home
- The patient dies
- The patient revokes his or her consent to participate in the study

### Strategies to improve adherence to interventions {11c}

The specialized nurses take care of the PwDs and their individual needs and will handle all interventions and monitor the completion or incompletion of all tasks frequently. To improve the adherence, the specialized nurses are continuously in and close contact with the PwD during the intervention.

### Relevant concomitant care permitted or prohibited during the trial {11d}

The InDePendent study is a pragmatic trial, which aims to implement DCM with extended nursing roles under routine care conditions. Concomitant care is permitted during the trial and will be documented as accurately as possible.

### Provisions for post-trial care {30}

Not applicable

### Outcomes {12}

The primary outcome of the study is the number of unmet needs in the intervention group compared to the waiting-control group 6 months after baseline. Unmet needs will be assessed with the German version of the Camberwell Assessment of Need for the Elderly (CANE). CANE is available in two versions: one for the PwD (if there is no participating caregiver) and one for the informal caregiver (if the caregiver participates in the study). The result of the CANE is a sum of open and unmet needs.

Secondary outcomes include:

1. Quality of life

The Quality of Life in Alzheimer’s Disease (Qol-AD) will be used to assess participants’ and caregivers’ quality of life [41]. The German version of the Qol-AD is a well-validated instrument for assessing HRQoL in patients suffering from different dementia diseases, especially for those being mild to moderately cognitively impaired [42].

2. Caregiver burden

Caregiver burden will be assessed by using the Zarit Burden Inventory [43].

3. Acceptance of the care model (process evaluation—only in the intervention group)

In addition to the quantitative analyses, we will perform semi-structured, qualitative interviews evaluated by application of qualitative content analysis [44].

4. Overall health status and economic analyses

For the health economic evaluation, health-related quality of life measured by the EQ-5D-5L and the utilization of healthcare services and informal care measured by the Resource Utilization in Dementia Questionnaire (RUD) [45] and the Questionnaire for Health-Related Resource Use in an Elderly (FIMA) [46] will be assessed.

### Sample size {14}

The primary endpoint of this study is the number of unmet needs. Recent literature points out that PwDs have on average 1.87 unmet needs with a standard deviation of 2.0 [47]. The sample size calculation was based on an assumed reduction of the number of unmet needs of 35% and a randomization ratio of 1:2 (1: intervention group, 2: waiting list). In a previous study, where dementia care management was also carried out, and patients were recruited from GP practices, there was an intra-cluster correlation (ICC) of 0.16 with a variance of ICC of 0.06 and a cluster size of five (five patients per GP practice). This ICC, variance of ICC, and cluster size were also used to calculate the sample size for this study. Therefore, a sample size of *n* = 345 PwD and 69 clusters would be needed to demonstrate an implicit effect of 1.22 unmet needs in the intervention group compared to 1.87 unmet needs in the control group at a significance level of *α* = 0.05 and statistical power of 80%. A loss to follow-up of about 30% was assumed due to the elderly population and the inclusion of a waiting-list group. Overall, this requires the recruitment of *n*=465 participants of 93 clusters to demonstrate a significant intervention effect. Therefore, a total of *n* = 310 patients in the waiting-control group (recruited from 62 GP practices) and *n* = 155 patients in the intervention group (from 31 GP practices) will be necessary to detect a significant difference between the intervention and the waiting control group concerning a 35% reduction of unmet care needs.

The randomization ratio of 1:2 was used due to the higher probability of dropouts in the waiting control group, which is predominantly a problem in times of the COVID-19 pandemic and the introduced lockdowns, contact restriction and the high risk of severe COVID-19 progressions, subsequent hospitalization, and death in the elderly population.

### Recruitment {15}

Participants will be recruited by participating GPs or specialists (neurologists, psychiatrists) practices in the participating physician’s networks. To identify community-dwelling patients, GPs approach their patients who already have received a formal dementia diagnosis and systematically screen patients at 70 and above for dementia using the DemTect instrument [48]. People who meet all inclusion criteria will be informed by their GP in detail about the study and invited to participate. Patients who provide written IC to participate will subsequently be included in the study.

## Assignment of interventions: allocation

### Sequence generation {16a}

Five physician networks participate in the study. In each network, participating practices are randomized following a 1:2-format to either the intervention- or waiting-control group. Block randomization will be computer-generated and operated in blocks size six using RandList software (Version 2, Datinf, Germany).

### Concealment mechanism {16b}

The allocation list is concealed by access restrictions. Only staff of the project evaluation team has access to the allocation list. Non-authorized staff cannot access the allocation list.

### Implementation {16c}

GPs were allocated based on a 1:2 (intervention:waiting list) cluster-level block randomization. The assignment list at the cluster level with defined assignments to the groups was determined prior to physicians’ assignment. The assignment of GPs was implemented when the first patient provided written informed consent in a GP practice. Physicians, therefore, filled in the next available slot on the assignment list, either the waiting list or the intervention group, when the study center received the informed consent documents. Thus, physicians’ participation decisions were made independently and prior to their group assignment.

The allocation sequence will be performed by the evaluation team using RandList software.

## Assignment of interventions: blinding

### Who will be blinded {17a}

Due to the nature of the intervention in this study, it is not possible to blind the study staff (nurses or practitioners) or the participants. Physicians and patients will become aware of their randomization status throughout the course of the study. The study is not blinded.

### Procedure for unblinding if needed {17b}

Not applicable.

## Data collection and management

### Plans for assessment and collection of outcomes {18a}

After study inclusion, all participants will receive a baseline assessment in their homes carried out by study nurses in the waiting-control group and dementia specialized nurses in the intervention group. Follow-up assessments will be carried out 6 months after the baseline assessment by the dementia specialized nurses in both study groups.

The primary outcome participants’ unmet needs will be assessed by using the Camberwell Assessment of Need for the Elderly (CANE). It consists of 27 categories (including two categories for the caregiver), which assess participants’ needs in various domains (e.g., living situation, household, nutrition, personal hygiene) followed by a question, whether the participant has an unmet need in this area (yes, no) [49]. CANE has been determined suitable for scientific use in dementia and demonstrates appropriate criterion validity [50]. It can easily be used by a wide range of professionals without formal training.

Secondary outcomes include (i) the Quality of Life in Alzheimer’s Disease (Qol-AD), a 13-item questionnaire designed to provide both a patient and a caregiver report of the quality of life. Each QoL-AD item is rated on a four-point scale, ranging from poor (1) to excellent (4). Results of the Qol-AD can be summarized in a score ranging from 13 to 52, in which higher numbers indicate higher quality of life [51]. (ii) Caregiver burden will be assessed by using the Zarit Burden Inventory, a caregiver self-report 22-item questionnaire. Each item on the interview is a statement which the caregiver is asked to rate using a 5-point scale. Response options range from 0 (never) to 4 (nearly always). The result of the instrument is a sum between 22 and 88, higher results indicate a higher subjective level of burden [43]. (iii) In addition to the quantitative analyses, we will perform qualitative interviews to assess the acceptance and satisfaction of the innovative care concept from different perspectives: people with dementia, caregivers, DCMs, and GPs. Qualitative data will be evaluated using content analyses [44]. (iv) Health economic analyses will assess the cost-effectiveness of the dementia care management compared to usual care by using the EQ-5D-5L [50], RUD (Resource Utilization in Dementia Questionnaire) [45], and FIMA (Questionnaire for Health-Related Resource Use in an Elderly Population) questionnaires [46]. The EQ-5D-5L is a standardized generic instrument for measuring health status. It has been widely used in population health surveys, clinical studies, and economic evaluation, measuring patients’ health utility in five dimensions: mobility, self-care, usual activities, pain/discomfort, and anxiety/depression. Health utilities will be combined with patients’ mortality as quality-adjusted life years by using the area under curve method. The RUD [45] and FIMA [46] instruments will be used to determine the utilization of informal care and caregivers’ productivity losses and the utilization of healthcare services from a payer’s perspective, respectively.

### Plans to promote participant retention and complete follow-up {18b}

The DCM will be in frequent contact with both the GPs and the PwD and their relatives. They can address any upcoming questions and concerns. We will provide internal trainings, newsletters, information events, and regular project meetings to maintain the awareness and motivation of all study partners. All changes including revocation of consent or discontinuation of participation will be documented in the study software.

### Data management {19}

Study nurses (waiting-control group) and dementia specialized nurses (intervention group) will collect data by usage of a touch-screen tablet during face-to-face interviews with study participants. Data will be documented in electronic case report forms (eCRFs), including automatic plausibility and completeness checks. All data stored locally on the tablet will be encrypted. The Java-based documentation system is based on the concept of offline clients. Each user (i.e., study nurses and DCMs) has an individual login. The client-server stores all data in the project data management system [52]. Secure data transfer from the tablets to the central database will be ensured by the application of virtual private network technology (VPN).

### Confidentiality {27}

Personal data will be stored separately from participants’ health data. Data storage is managed according to current standards for data security and data privacy, which is documented in the data protection policy by the Institute for Community Medicine. Only the study nurses and DCMs have access to personal data during baseline and follow-up examinations. Paper documents (i.e., IC) will be stored in a secured way. Only authorized persons can access these documents.

Access to the study software based on individual user passwords and a detailed right and role system in which data minimization and security are priorities. The study nurses and DCMs will only be able to review participants and GPs in their personally assigned study region.

### Plans for collection, laboratory evaluation, and storage of biological specimens for genetic or molecular analysis in this trial/future use {33}

Not applicable.

## Statistical methods

### Statistical methods for primary and secondary outcomes {20a}

All statistical analyses will be conducted using pseudonymized data. Initially, primary and secondary outcomes will be analyzed with descriptive analyses. At baseline, the randomization procedure will be verified by comparison of the intervention- and waiting-control group based on descriptive statistics for various variables (randomization check). Should significant differences between the intervention and waiting-control group be identified, analyses will be adjusted for relevant variables. Quantitative parameters will be evaluated using hierarchical multi-level analyses to consider the study design with cluster randomization using physician’s practice and physician’s networks as context variables.

Response rates will be calculated for each group at each time point of analysis and compared between groups. Due to the expression (data count variable) of the primary outcome (unmet needs), a (multi-level) Poisson regression model based on the intention-to-treat principle will be fitted.

Respective to the secondary outcomes, analyses will apply linear or logistic regressions. Concerning the within-trial cost-effectiveness analysis, the incremental cost-effectiveness ratio (ICER) will be calculated by application of the incremental cost per QALY (Quality-adjusted life years) gained by the intervention compared with the waiting-control group. Descriptive statistics will be used to demonstrate unadjusted incremental cost and QALY. Nonparametric bootstrap resampling will be used to handle sampling uncertainty in the ICER. The probability of cost-effectiveness will be calculated by the application of different willingness-to-pay (WTP) margins based on the net monetary benefit approach [53]. All analyses will be performed with the statistical software IBM SPSS Statistics (version 26.0.0.0. or later, IBM Corp., NY, USA) and STATA (version 16 or later, StataCorp. LLC, TX, USA).

## Qualitative analyses

The evaluation of the trial follows a mixed-methods approach. In addition to the quantitative analyses, we will conduct structured and guided interviews with experts who are involved in the care process (e.g., physicians in the primary care setting). Interviews will be recorded and then transcribed verbatim / partially by application of MAXQDA software (version 12.0 or later, VERBI, Berlin, Germany). Analysis of the transcribed material will follow a qualitative content analysis approach [42]. Results of both, quantitative and qualitative data will be analyzed in combination regarding patient-relevant endpoints and process evaluation. The process of the evaluation follows the guidelines of the Medical Research Council 2006 [54].

### Interim analyses {21b}

Not applicable.

### Methods for additional analyses (e.g., subgroup analyses) {20b}

In addition to the intention-to-treat analyses, we will perform subgroup analyses with participants who received the intervention (per-protocol analyses). Further analyses will be performed with specific subgroups of the study sample (e.g., severity of dementia, different home care situations).

### Methods in analysis to handle protocol non-adherence and any statistical methods to handle missing data {20c}

In univariate and bivariate analyses, the number and distribution of missing data will be identified. In multivariable analysis, missing data will be handled using multiple imputation via chained equations, stratified for the respective group.

### Plans to give access to the full protocol, participant-level data, and statistical code {31c}

Information will be provided on request.

## Oversight and monitoring

### Composition of the coordinating center and trial steering committee {5d}

The DZNE is the coordinating center for the InDePendent study. All partners involved in the study will report regularly to the DZNE on the progress within the study. The ICM provides its own team for the objective evaluation of the study results.

### Composition of the data monitoring committee, its role, and reporting structure {21a}

The study is a population-based cluster randomized controlled trial evaluating the efficacy of a multi-component non-invasive intervention in people with dementia with a very low health risk for study participants. For this reason, a data monitoring committee (DMC) is not needed for our study. However, the authors have no competing interests and the results of the study are independent from the sponsor. All study results will be published.

Objective evaluation of the study is conducted by the Institute of Community Medicine, which has an external and independent role to the consortium leader of the trial, the German Center for Neurodegenerative Diseases e.V.

### Adverse event reporting and harms {22}

We will assess and document all adverse events and unintended effects, and communicate them to relevant study participants. However, we consider the specific risks for participating PwD to be very low. No negative effects on the quality of life of PwD as well as disability or other undesired events due to the intervention are to be expected which is supported by previous studies involving DCM interventions [55].

Nonetheless, it could be possible that some participants feel harassed or pressured by the intervention or the repeated contact attempts. To detect possible adverse events, participants will be asked by questionnaires throughout the study as part of the process evaluation.

### Frequency and plans for auditing trial conduct {23}

Not applicable

### Plans for communicating important protocol amendments to relevant parties (e.g., trial participants, ethical committees) {25}

We will communicate protocol modifications and relevant process changes to the Local Ethical Committee at the University Medicine Greifswald and to all relevant ethical committees of the study partners. All changes will be noted in the study registration as well.

### Dissemination plans {31a}

We will publish all relevant study results in scientific journals to share our results with the scientific community and allow for scientific discussion. In addition, all study results will be communicated at scientific meetings and conferences by the investigators. Authorship will be shared between persons involved in the study following the current guidelines of the International Committee of Medical Journal Editors (ICMJE). We plan to communicate the study results to the study participants and health service providers. In addition, results will be used for designing and parameterizing further research projects.

## Discussion

The rapid increase of the number of PwD and the existing and growing shortage of primary care physicians are a challenge for health care systems in many countries in the next decades, requiring new and innovative models of care, especially in rural and remote areas [1, 3–5]. Within the InDePendent project, a new model of advanced nursing care will be developed and tested under routine care conditions in the primary care setting of physicians’ networks. Our study aims to identify unmet nursing, medical, psychosocial, pharmaceutical, and social needs and to improve the living and care situation of people with dementia and their relatives through a redistribution of tasks between specialized nurses and primary care physicians.

The results of the InDePendent study will provide evidence on how to reduce unmet needs among people with dementia. This study will provide evidence for both relevant stakeholders and health policy makers. Trial results will provide evidence for the development of a legal framework for innovative care concepts in the primary care setting, which has an eminently important role for the care of PwD. Particularly the study has the potential to introduce an innovative care concept based on cooperation and the redistribution of tasks between specialized nurses and primary care physicians into regular health care in Germany.

The results of the study will help to modify existing guidelines in dementia. Finally, the project aims to relieve the burden on primary care physicians and to expand the role of nurses, who will assume certain tasks, usually carried out by primary care physicians. This could help to increase professionalization in order to render nursing more attractive for young people as well as increase the motivation to stay in the job for more experienced colleagues. Both are urgently needed to correspond to the growing healthcare challenges resulting from the demographic change. Since the trial will be conducted under real-life conditions in the primary care setting, external validity will be high and the results of the trial are likely generalizable to the primary care setting in other regions.

Due to COVID-19, recruitment took place under difficult conditions. In order to avoid personal contact during the study, a telephone or online video-conference-based survey is offered as an alternative method for data collection.

## Trial status

This is the protocol version 2.0, 2 May 2021. Enrolment into the study started on 1 January 2021 and is estimated to continue until the end of June 2022.

## Data Availability

Information will be provided on request.

## Abbreviations

DCM: Dementia care management
GP: General practitioner
VPN: Virtual Private Network
ICM: Institute for Community Medicine
IMS: Intervention-management-system
PwD: People with dementia
ANP: Advanced nursing practice
